# The path for medical associations to sponsor trustworthy guidelines: is it feasible?

**DOI:** 10.1177/01410768221095240

**Published:** 2022-04-29

**Authors:** Domenico Pagano, Philip Home, Alar Irs, Jonathan Ledermann, Giuseppe Curigliano, Tsuguo Iwatani, John Mandrola, Nick Freemantle

**Affiliations:** 1 University Hospitals Birmingham, Birmingham, B15 2GW, UK; 2 Translational and Clinical Research Institute, Newcastle University, NE1 7RU, UK; 3 Heart Clinic, Tartu University Hospital, 50406 Tartu, Estonia; 4 UCL Cancer Institute, University College London, WC1E 6BT, UK; 5 University of Milano and European Institute of Oncology, IRCCS, 20122 Milan, Italy; 6 Breast Surgery, National Cancer Center Hospital East, 277-8577 Chiba, Japan; 7 Baptist Health Louisville, Louisville, KY 40207, USA; 8 Institute of Clinical Trials and Methodology, University College London, London, WC1E 6BT, UK

## Introduction

In an effort to support best practices, medical associations often develop clinical practice guidelines. This can come with significant drawbacks. Due to professional and scientific issues coming to light after publication, in 2019 the European Society of Cardiology (ESC) and the European Association for Cardiothoracic Surgery (EACTS) recalled their recent 2018 clinical guidelines for treatment of left main coronary artery stenosis.^1^

Recently we have seen a significant disparity between guideline recommendations on aortic valve stenosis from medical associations^2, 3^ and the National Institute for Clinical Excellence (NICE).^4^ Here association guidelines promote the use of transcatheter aortic valve implantation as an equivalent to surgery, while NICE recommends surgery as a first-line treatment. Thus, the same clinical evidence has led to substantially different recommendations.

These two issues have renewed concerns as to whether medical associations are reliable authors of clinical guidelines.^5^ There is broad literature identifying the shortfalls of guidelines written by associations, issues remaining largely unresolved. In this commentary, we provide an insider’s view of these problems and suggest steps to address them. The overall principles for writing clinical guidelines (WHO and IOM) describe^6^ important areas for associations to address the most relevant being: management of conflicts of interests (COIs) and methodological transparency evidence appraisal.

## Guidelines, funding and bias

The inadequacy of managing organisational and personal duality of interests is a longstanding issue. Associations contribute to improving healthcare in the area of new technologies through educational meetings and training. Content expertise comes with the advantage of clinical knowledge but it also brings diverse personal and corporate COIs (Table 1) which challenge the validity and trustworthiness of their guidelines.^6, 7^ Transparency and full declaration of COIs, while necessary, are not sufficient to ensure bias is neutralised, as Goldberg has discussed persuasively.^8^

**Table 1.** List of potential sources of bias in association guidelines.

1.	**Academic bias:** from individuals with a belief stake in findings to which they have contributed intellectually (and which may be the source of their future status).
2.	**Clinician bias:** from individuals whose practice funding depends on healthcare delivery.
3.	**Guidelines' developer bias:** from industrial funding of the guidelines themselves.
4.	**Guidelines' developer bias**: from personal and institutional funding of specialists concerned, leading to halo effects.
5.	**Patient-input bias:** through insistence on a right to the latest technology even when unproven and/or not cost effective.
6.	**Healthcare funder bias**: due to concern over affordability of new approaches, or through the lobbying of government by strategically important industry.
7.	**Political bias**: from governments believing political capital can be gained by taking certain positions.
8.	**Association bias:** often related to point 2, 3 and 5 above, but including industrial support for their activities, meetings and other activities.

The large financial commitment in the development of new therapies requires that capital investments bring early returns, with short-term studies for therapies indicated for long-term use a particular problem. Rapid decisions by regulators permitting early access to new technologies shift the responsibility for access to new treatments to those who reimburse technologies; decision makers who often make use of association guidelines. Timely recommendations may then not be robust. This is how COIs may have led to the disparity in judging the limited evidence for the recommended treatment of aortic stenosis.

Medical associations and industry often enjoy a symbiotic relationship. Industry benefits from clinical expertise and access to those who prescribe treatments, while medical associations benefit from financial support. Annual meetings for members are often largely supported by industry, through the purchasing of sponsored educational sessions and exhibition space, and the surplus is often the major source of for associations.^9^ Associations do not always accurately describe their ties with industry. Sometimes guideline funding is said to come from general funding (meaning it was not raised for the specific purpose of guideline production),^10^ and is often not described in detail. We suggest that associations should include details of individual industry relationships, including amounts and dates rather than consolidated under grouped financial income headings, as this level of transparency will enable the reader to comprehend the context in which a recommendation is made.

There are other interests that can contribute to bias when writing guidelines, as listed in Table 1, some non-financial. We speculate that the first two areas of COIs in the figure are the most important, seldom reported and the most difficult to mitigate. Associations often appear to lack robust process for the management of individual COIs.

It has become commonplace for content experts to play simultaneous and potentially conflicting roles first in the generation of evidence as trialists, second in codification of that evidence as guideline writers, and third as influential officials in medical associations playing a role in obtaining industry funding.^11, 12^ These overlapping roles can lead to a lack of self-appreciation of bias, and, at worst, may lead to unsafe guideline recommendations, as occurred in the joint ESC-EACTS myocardial revascularisation guidelines.^11–13^ An obvious and sometimes implemented solution is for trialists not to play a role in codifying practice in guidelines, but this tends to lead to exclusion of the very people who know the strengths and limitations of the evidence available. However, we believe that such assessments are better accomplished with a significant input from specialists in evidence-based critical appraisal with no connections to the evidence being appraised. The United States Preventive Services Task Force provides an example of an independent volunteer panel of national experts who make recommendations based on a rigorous review of existing peer-reviewed evidence.^14^

## Examples of current practice

There is heterogenicity in the approach to dealing with COIs among different organisations. In diabetes, of particular note are guidelines from the American Diabetes Association (ADA), European Association for the Study of Diabetes (EASD) and International Diabetes Federation. The ADA's annually updated Standards of Care are nominally funded from ‘general funds' without request for specific sponsorship, and the provenance of the writing is made clear.^10^ Funding for the regularly updated ADA/EASD consensus reports on glucose-lowering management is also stated as coming from general organisation resources, but the associations' sources of support are not made clear.

The three UK, European and global diabetes associations are all known to have relationships with industry for guideline and conference activities, and all recognise issues around individual experts having dualities of interests when working on guideline and consensus documents. However, this is handled by a requirement for transparency rather than exclusion of individuals either from guideline groups or more particularly from chairing or being lead writer on such activities. Transparency may identify the potential for bias, but does not neutralise it. However, transparency in this manner may enable the implementation of specific strategies to address bias. For example, the chair of a guidelines group could be excluded if they are found to have financial conflicts above a specific monetary value. There is a need to balance the potential for bias and expertise. For example, it may exclude too many with product knowledge if all potential guidelines group members are required to have no COIs. This predetermined guidance on the management of conflicts within an organisation producing and disseminating guidelines could also identify red flag areas where individuals with specific conflicts abstain from offering a judgement.

The costs of cancer treatment are significant for healthcare and so are the financial investments in researching new therapies. Thus, oncology practice guidelines are important for optimising treatment and introducing new costly therapies. The European Society for Medical Oncology (ESMO) guidelines are used worldwide to guide treatment decisions and assist healthcare payers in evaluating the benefit of new anti-cancer drugs. All authors must declare any potential COIs. A compliance committee verifies if COIs generate potential bias in the writing process. Activities and responsibilities of the committee include implementing and managing the disclosure of interest policy, and overseeing background checks.

The American Society of Clinical Oncology guidelines, which have a major impact on oncology practice worldwide, require the majority of panel members (51%), including the panel chair, to be free of relevant industry relationships.^15^ However, the issues with non-financial COIs (as illustrated in Table 1) and transparency about the source of funding remain.

It is not uncommon for ESC high-impact cardiovascular guidelines to be written and reviewed by members with significant and relevant industry financial ties.^16^ The 2021 ESC-EACTS Guidelines for the management of valvular heart disease^3^ were written by 21 authors, of whom 18 declare one or more financial COIs receiving unquantified income from industries (personal, institutional or both) whose products are included in the guidelines. Some authors declare owning shares or receiving royalties. Non-financial COIs were not reported, and the associations did not describe how these prevalent COIs were managed.

## Recommendations and conclusion

In the USA, the www.openpaymentsdata.cms.gov system, set up as a result of the Sunshine Act, provides some degree of transparency and validation of the payments declared by industry to physicians, although physicians often under-declare their income.^17^ In Europe there is no mechanism to validate declared payments; such a transparent system is urgently needed despite barriers of cooperation between countries.

The task of converting available clinical evidence into robust treatment recommendations is not straightforward. High-quality evidence from randomised controlled trials (RCTs) is often unavailable, or when present, burdened with internal validity challenges, such as bias in design, execution, statistical analysis and interpretation of results. Thus, guideline recommendations require judgement and subjectivity, an area where unconscious bias from COIs may have adverse influence. A clear and reproducible process for guideline production should be described and implemented, with the link between evidence and specific guideline recommendations made explicitly. We recommend that associations invest in designing and implementing suitable protocols which describe the entire process including: how members are recruited to the task; identifying roles and responsibilities; and defining how evidence-based recommendations are derived. These steps should be part of formal standard operating procedures (SOPs) to ensure best internal practice.

With regard to transparency of the process itself, we first recommend that all COIs of all members are described; this should include non-financial COIs as illustrated in Table 1. But importantly, in addition, we recommend that all the steps to achieve a recommendation are published including minutes of the meetings, voting results where applicable and evidence tables provided to the panels. Given that the majority of clinical recommendations are often subjective and not based on robust trial evidence, it is important for the reader to understand how these are achieved and their strengths and limitations.

While transparency about COIs and how they might influence guideline process is an important step, we believe that most biases cannot be easily mitigated.^18^ Associations should have pre-agreed procedures and guidelines on the management of COIs (financial and otherwise), which are known to introduce bias or perception of bias in the process and outcomes. When all COIs have been declared by potential members of the committees, we recommend that an independent compliance committee decides eligibility and to what extent participation is allowed against the pre-agreed SOPs. However, finding a true independent compliance committee can be challenging. It is important to recognise that the role of clinicians with significant COIs should be very limited, and they should be excluded from the process of shaping the final recommendations.

While the input of clinical experts remains important, the discussions of the guideline groups should be reported by independent scientific ‘writers' in the first instance, and can then be used to derive recommendations for editing and approval by all members. The role of formal organisations, such as GRADE or Cochrane collaboration,^19^ in the process may be enhanced. This will add independent methodological credibility and robustness, although presently their methodology struggles where the RCT evidence base is thin.

An independent accreditation process might be implemented to rate the quality of guidelines produced by associations against ‘defined best practice’. Medical journals should publish only guidelines that meet required standards. However, medical associations often own their medical journals and this might add further pressures and bias. Independent editors are not immune to the attractions of guidelines to readers and citation rates. Associations should also establish opportunities for training participants in guideline development methodology a step which will provide an investment in the future for members and will facilitate the necessary change in culture.

Medical associations must recognise the significant limitations of their current approaches and embrace the necessary changes. The steps described above will go a long way to the enhancement of the trustworthiness of the guidelines if implemented.

However, in our experience, even with a call for such reform, there are practical challenges about how to assure that proposed significant changes would in be adopted and adhered to. Thus, short of professional associations no longer writing or sponsoring clinical guidelines, the challenges described may no-matter the specific reforms that are recommended, and perhaps implemented. Exemplars, such as The Conference on Harmonisation, have demonstrated how appropriate regulation can improve the integrity of research conduct. Embracing appropriate external standards could similarly lead to an improvement in the trustworthiness of guidelines.

## Declarations

**Competing Interests:** The author(s) declared the following potential conflicts of interest with respect to the research, authorship, and/or publication of this article: DP is former Secretary General of the EACTS. EACTS receives grants and financial support from several pharma and medical device industries. DP has no financial disclosures. He was International Director, Society of Thoracic Surgeons – USA (2018–2021). PDH has led on many clinical trials and meta-analyses, and had lead roles in national (UK) and international guidelines. He has had personal and institutional funding for his research, lecturing and advisory activities from most major industrial partners in diabetes therapy and diagnostics. He advises national regulatory bodies in the UK, and reviews grant applications for Diabetes UK. AI is a board member at the Estonian Society of Cardiology which as an organisation receives funding for educational and research activities from most major cardiovascular pharmaceutical and technology companies. He is a staff member of the Estonian national medicines’ regulator, a member of the Estonian National Guideline Advisory Board (which uses GRADE methodology) and of the Committee for Human Medicinal Products of the European Medicines Agency. The views expressed in this article are the personal views and may not be understood as being made on behalf of or reflecting the position of the European Medicines Agency. JL is vice-president of ESMO and editor of ESGO Gynaecological Cancer Guidelines; has received consultancy, advisory board and speakers fees from AstraZeneca, Tesaro-GSK, Clovis Oncology, Artios Pharma, Regeneron, Pfizer, Eisai and NeoPharm; his Institution has received grants from AstraZeneca and Merck/MSD. TI and JM report no duality of interests. NF has been involved in the design and analysis of many clinical trials and meta analyses/network meta analyses. Through a grant to his institution, he advises EACTS on methodological questions and provides educational input, contributing to the development of several guidelines including collaboratively with other medical associations. He has provided advice to a number of manufacturers of drugs and devices including Abbott Singapore, Aimmune, ALK, AstraZeneca, Grifols, Galderma, Ipsen, Novatis, Sanofi Aventis and Gilead.

**Funding:** None declared.

**Ethics approval:** Not applicable.

**Guarantor:** DP.

**Contributorship:** All authors contributed to the drafting, revision and approval of this document. The views expressed are their own, and do not seek to express in any way the views of any organisation with which they are associated.

**Acknowledgements:** None.

**Provenance:** Not commissioned; peer-reviewed by Renato Ferreira da Silva and Rupasvi Dhurjati.

**ORCID iDs:** Jonathan Ledermann https://orcid.org/0000-0003-3799-3539 Nick Freemantle https://orcid.org/0000-0001-5807-5740
